# Supplementary material for: Magnetically Assisted Drug Delivery of Topical Eye Drops Maintains Retinal Function In Vivo in Mice
Source: Pharmaceutics. 2021 Oct 9;13(10):1650. doi: 10.3390/pharmaceutics13101650 (PMC8540400; doi:10.3390/pharmaceutics13101650)
Supplement: Supplementary file 1 [file pharmaceutics-13-01650-s001.zip › pharmaceutics-1380528 - supp corrected.pdf]

pharmaceutics

MDPI

Article

Magnetically Assisted Drug Delivery of Topical Eye Drops Maintains Retinal Function In Vivo in Mice

Marco Bassetto, Daniel Ajoy, Florent Poulhes, Cathy Obringer, Aurelie Walter, Nadia Messadeq, Amir Sadeghi, Jooseppi Puranen, Marika Ruponen, Mikko Kettunen, Elisa Toropainen, Arto Urtti, Hélène Dollfus, Olivier Zelphati and Vincent Marion

Table S1. elution protocols used for Guanabenz (orange) and Valproic acid (blue) quantification with HPLC analytical equipment.

HPLC Elution Protocols			
Guanabenz (GBZ)		Valproic acid (VPA)	
Time (min)	Methanol (%)	Time (min)	Methanol (%)
5	30	6	30
5–8	100	6–8	100
8–13	100	8–15	100
5 post run	30	5 post run	30

Table S2. organization of the groups and number of animals used for in vivo assessment of magnetic-assisted GBZ and VPA delivery to the photoreceptors.

Experiment	Mice Model	Treatment	Number of Animals
MNPs ocular distribution (MRI)	Bbs$^{+/+}$	Untreated<br>NP01+NP04 (magnet)<br>NP01+NP04 (without magnet)	3 per group
Assesment of MNPs-based eyedrops safety	Bbs$^{+/+}$	Untreated	5 animals
		1 mg/ml loaded MNPs (NP01+NP04)<br>40 μg/ml loaded MNPs (NP01+NP04)<br>20 μg/ml loaded MNPs (NP01+NP04)	6 per group
Assessment of GBZ and VPA delivery in photoreceptors of *Bbs*$^{-/-}$ mice	Bbs$^{-/-}$	Untreated	5 animals
		40 μg/ml loaded MNPs (NP01+NP04)<br>40 μg/ml unloaded MNPs (NP01+NP04) control group<br>40 μg/ml loaded GBZ MNPs (NP01+NP04) control group<br>40 μg/ml loaded VPA MNPs (NP01+NP04) control group<br>GBZ + VPA	6 per group

Table S3. experimental groups and the exact genetic background of the mice used in each experiment.

Experiment	Treatment group	*Bbs* Model	Number of animals used
MNPs ocular distribution (MRI)	All mice	*Bbs*$^{+/+}$	9
Assesment of MNPs-based eyedrops safety	All mice	*Bbs*$^{+/+}$	18
Assessment of GBZ and VPA delivery in photoreceptors of *Bbs*$^{-/-}$ mice	Untreated group	*Bbs10*$^{-/-}$	1
		Bbs12$^{-/-}$	5
	40 μg Fe/ml loaded MNPs (NP01+NP04	*Bbs10*$^{-/-}$	1
		Bbs12$^{-/-}$	5
	GBZ + VPA solution	*Bbs1*$^{M390R/M390R}$	2
		Bbs12$^{-/-}$	4
	40 μg FE/ml unloaded MNPs (NP01+NP04) control group	*Bbs12*$^{-/-}$	6

40 µg Fe/ml loaded GBZ MNPs (NP01+NP04)	*Bbs10*-/-	4
control group	*Bbs12*-/-	2
40 µg Fe/ml loaded VPA MNPs (NP01+NP04)	*Bbs10*-/-	2
control group	*Bbs12*-/-	4

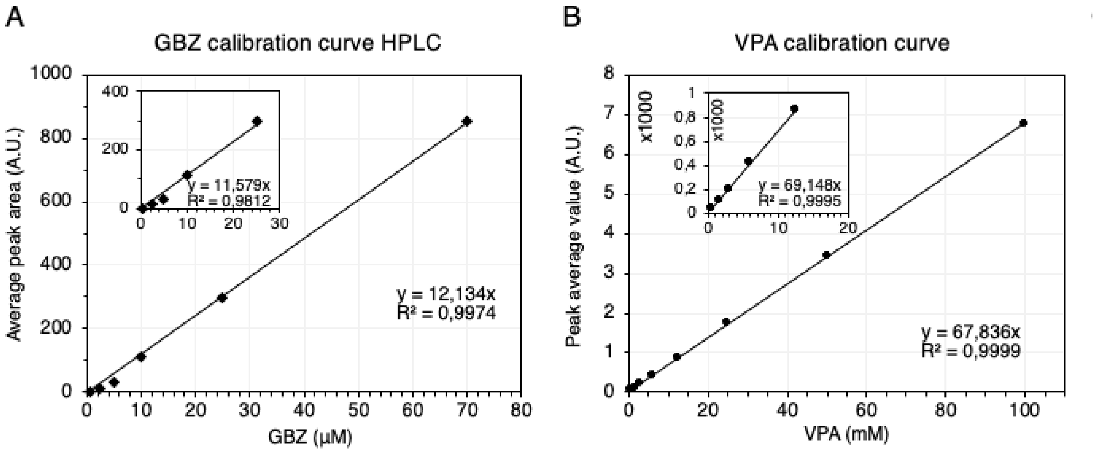

Figure S1. calibration curves established for guanabenz and valproic acid using HPLC. **(A)** Linear response for guanabenz (GBZ) in the range of 0.5–70 µM, the smaller graph shows the linearity at lower concentration 0.5–25 µM. **(B)** Linear response for valproic acid (VPA) in the range of 0.5-100mM, the smaller graph shows the linearity at lower concentration 0.5–15 mM.

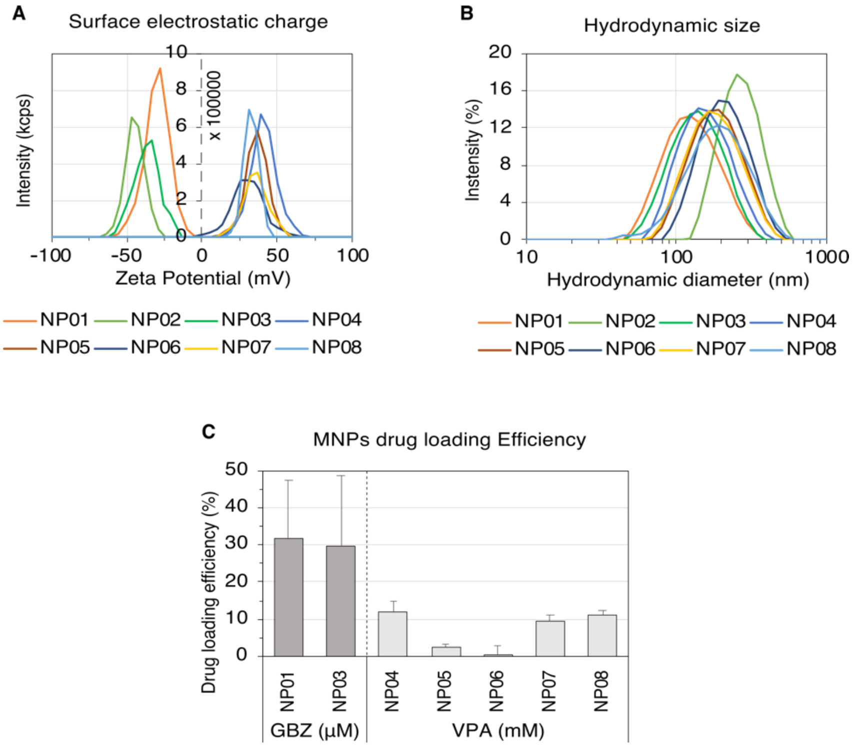

Figure S2. screening of MNPs used for GBZ and VPA loading via electrostatic interactions. Plots of the surface electrostatic charge (Zeta potential) **(A)** and hydrodynamic size **(B)** distributions. **(C)** NP01 is the best anionic MNPs to load GBZ (LE=31.5 ± 15.7%) and NP04 is the best to load VPA (LE=11.81 ± 3.14%), NP02 was excluded because it precipitated as soon as GBZ was added; results are presented as the mean of the values calculated from three independent sets of experiments each repeated three times and are expressed as % of drug loaded on the MNPs surface, error bars correspond to the standard deviation obtained from the average values calculated for each set of measurements, *n* = 9. Abbreviations: drug loading efficiency (LE).

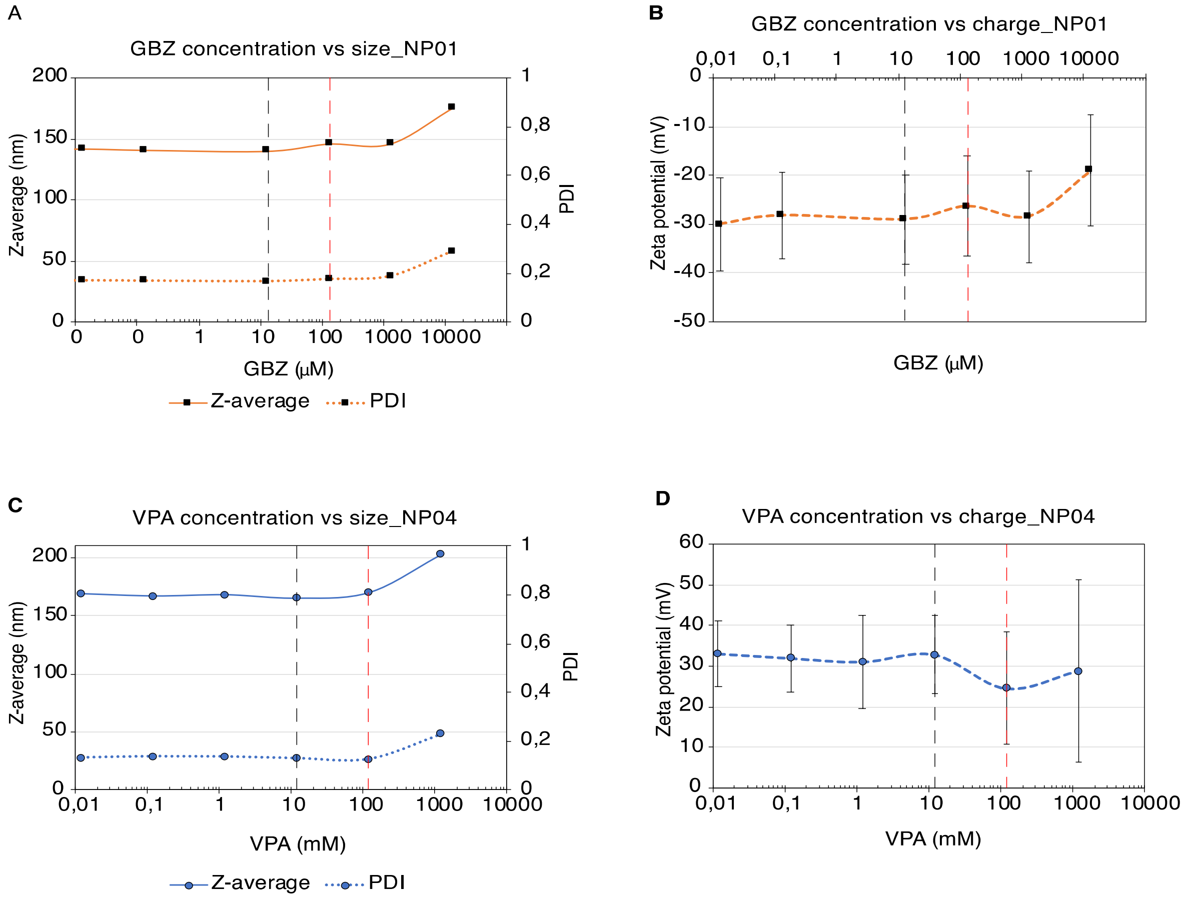

Figure S3. effect of GBZ and VPA concentration on the stability of NP01 and NP04 respectively by DLS and electrophoretic light scattering. **(A)** The values of NP01's average hydrodynamic size (Z-average) (orange continuous plot) and PDI (orange dotted plot) increase for GBZ concentrations higher than 15 μM (black dashed line), meaning that NP01 aggregates (red dashed line). **(B)** the plot of NP01's surface charge (Zeta potential) is in agreement with the measurements of both Z-average and PDI, hence confirming NP01 aggregation for GBZ concentration higher than 15 μM. **(C)** The values of NP04's Z-average (blue continuous plot) and PDI (blue dotted plot) increase for VPA concentrations higher than 12 mM (black dashed line), meaning that NP04 aggregates (red dashed line). **(D)** the plot of NP04's Zeta potential is in agreement with the measurements of both Z-average and PDI, hence confirming NP04 aggregation for VPA concentration higher than 12 mM.

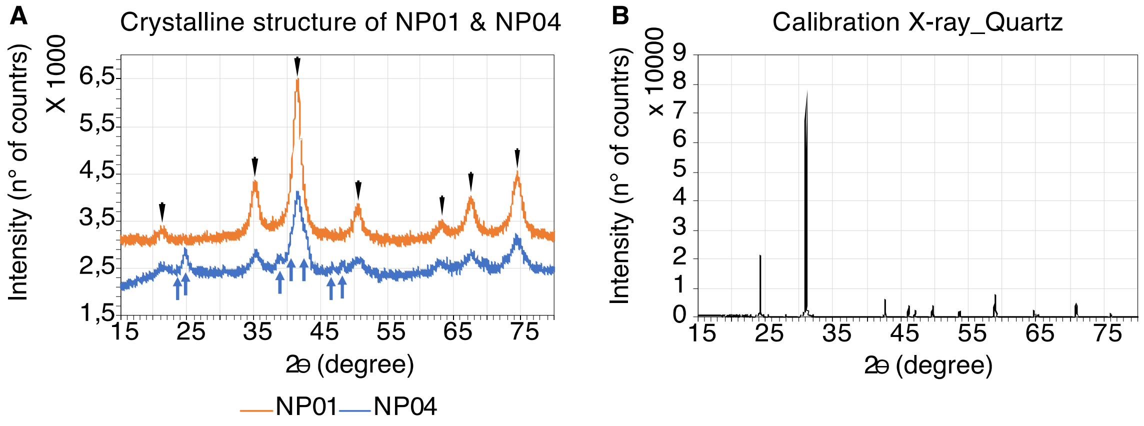

Figure S4. characterization of NP01 and NP04 by powder X-ray diffraction. **(A)** The diffraction pattern of both NP01 (orange plot) and NP04 (blue plot) contain seven strong diffraction peaks at Bragg angle (2θ) of 21,4°, 35,2°, 41,5°, 50,7°, 63,5°, 67,6° and 74,5° that correspond to the diffraction pattern of magnetite (black arrows), but in the diffraction pattern of NP04 there is also a minimal

contribution of goethite (blue arrows). Additionally, both diffraction patterns show the characteristic peak enlargement due to the nano-size range of the magnetic nano-cores compared to the peaks **(B)** obtained from quartz crystals of millimetric size. The time of exposure and geometric configurations are the same in both MNPs and quartz analysis that are carried out using Co Kα.

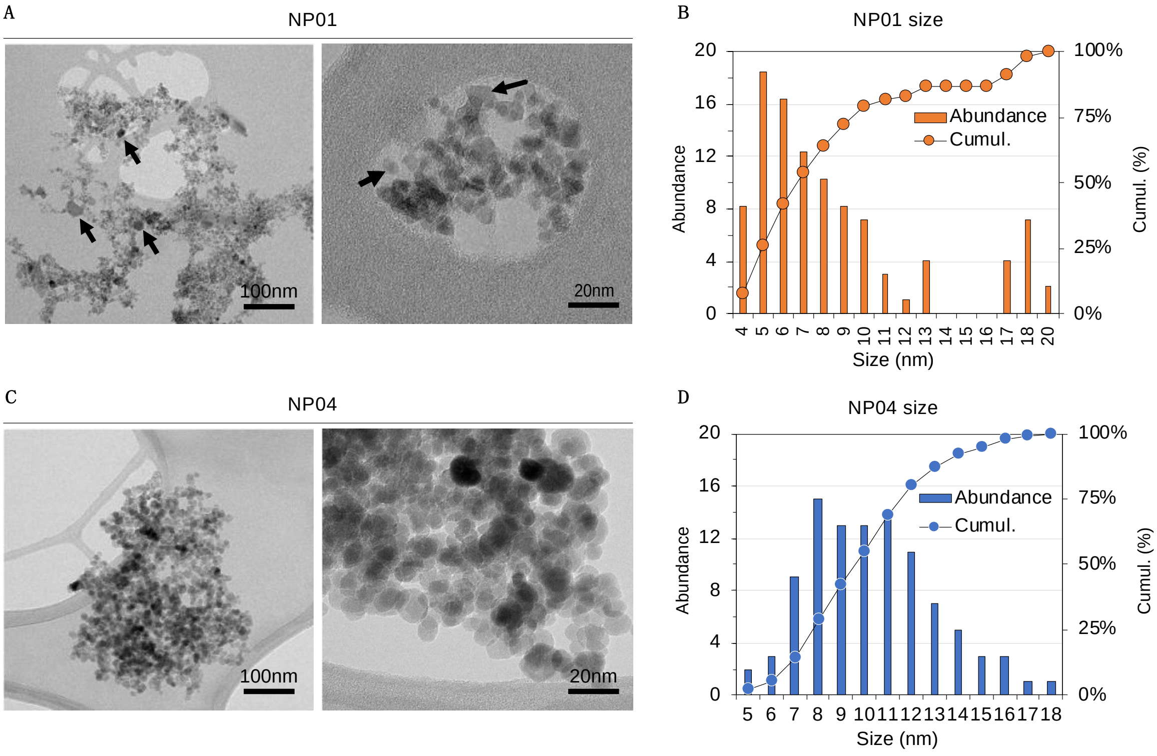

Figure S1: Characterization of NP01 and NP04 by TEM. Representative TEM images of NP01 magnetic nanocrystals showing both single round objects packed in agglomerates of larger size **(A)**. The magnetic crystals of NP01 have average size of 8 nm calculated from 100 single objects **(B)**, relative abundance (orange bars) and cumulative sizes (solid line). **(C)** Representative TEM images of NP04 showing magnetic nanocrystals as single round objects found in compacted agglomerates of larger size. The magnetic crystals of NP04 are more polydisperse as compared to NP01 and they also have average size of 10 nm calculated from 100 single objects **(D)**, relative abundance (blue bars) and cumulative sizes (solid line).

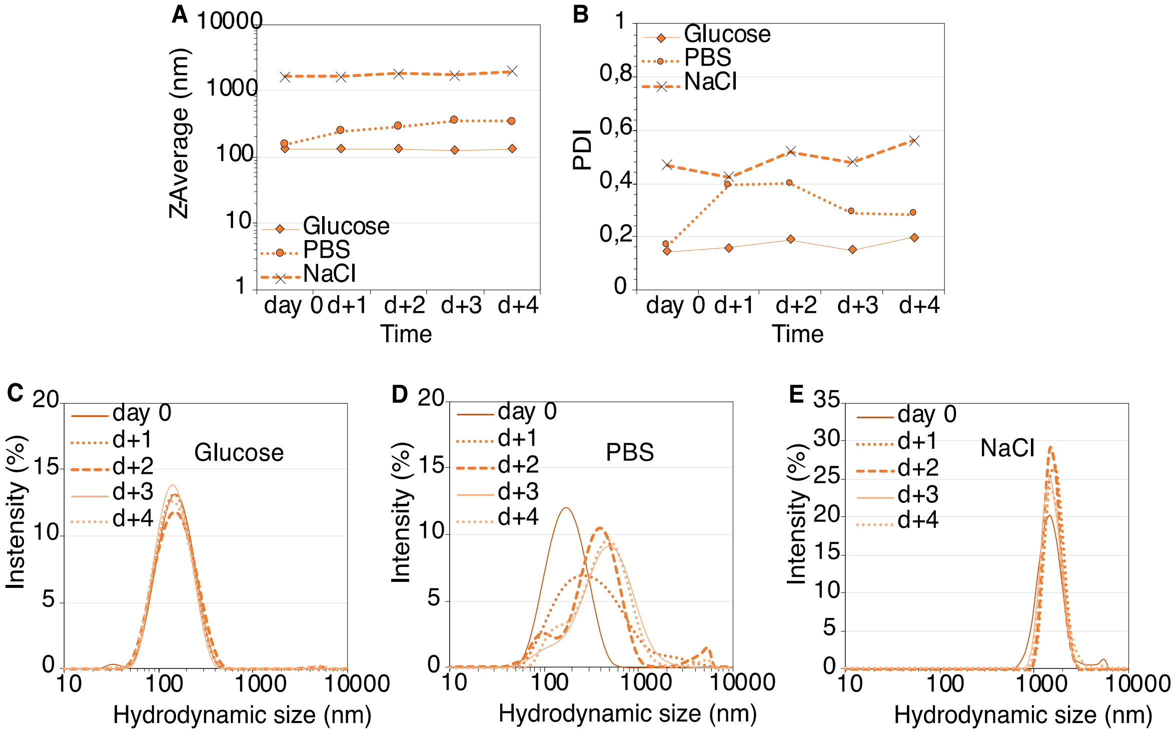

Figure S6. stability of NP01@GBZ in different vehicles by DLS technique. The values of both average hydrodynamic size (Z-average) **(A)** and polydispersity index (PDI) **(B)** do not increase in glucose (continuous line) during four days (d+4); instead the two parameters increase in PBS (dotted line) and NaCl (dashed line) as a consequence of aggregation. In glucose, the plots of the hydrodynamic size of NP01@GBZ **(C)** are characterized by a single peak of average hydrodynamic size of 164±114nm that remains unchanged up to d+4. **(D)** In PBS, the plots of the hydrodynamic size of NP01@GBZ right after dilution (day 0) show a single peak but the MNPs eventually aggregate in the next days as showed by the simultaneous broadening of the most intense peak and the appearance of secondary peaks. **(E)** In NaCl, NP01@GBZ aggregates in NaCl **(E)** right after dilution (day 0) as shown by the simultaneous localization of the peaks at micrometric values of hydrodynamic size and the significant increase of the light scattering intensity.

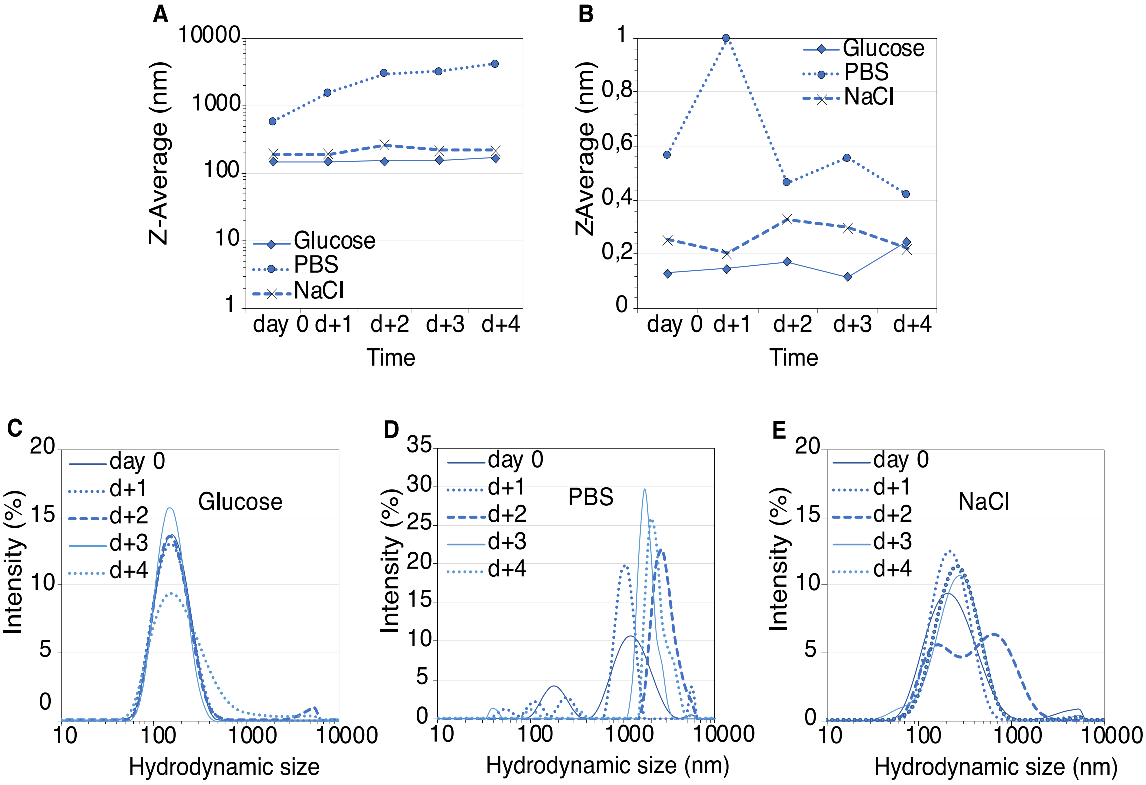

Figure S7. stability of NP04@VPA stability in different vehicles by DLS technique. The values of both average hydrodynamic size (Z-average) **(A)** and polydispersity index (PDI) **(B)** do not increase in glucose (continuous line) during four days (d+4); instead the two parameters increase in PBS (dotted line) and NaCl (dashed line) as a consequence of aggregation. **(C)** In glucose the plots of the hydrodynamic size of NP04@VPA are characterized by a single peak of average hydrodynamic size of 152 ± 127nm that remains almost unchanged up to d+4. **(D)** In PBS, NP04@VPA aggregates as shown by the simultaneous broadening and appearance of secondary peaks in the plots of the hydrodynamic size. **(E)** In NaCl, NP04@VPA is more stable than in PBS but it eventually aggregates during four days as shown by the broadening of the most intense peak and appearance of a secondary peak at d+4 in the plots of the hydrodynamic size.

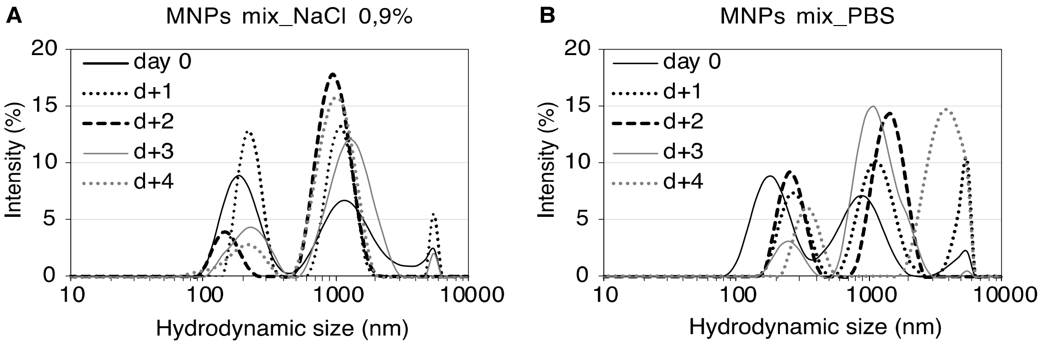

Figure S8. temporal evolution of hydrodynamic size of MNPs mix (NP01+NP04) loaded with drugs (GBZ and VPA) in vehicles containing electrolytes by DLS. The MNPs mix aggregates rapidly when NaCl **(A)** and PBS **(B)** are used as vehicle as shown by the appearance of multiple peaks located at micrometric values of hydrodynamic size.

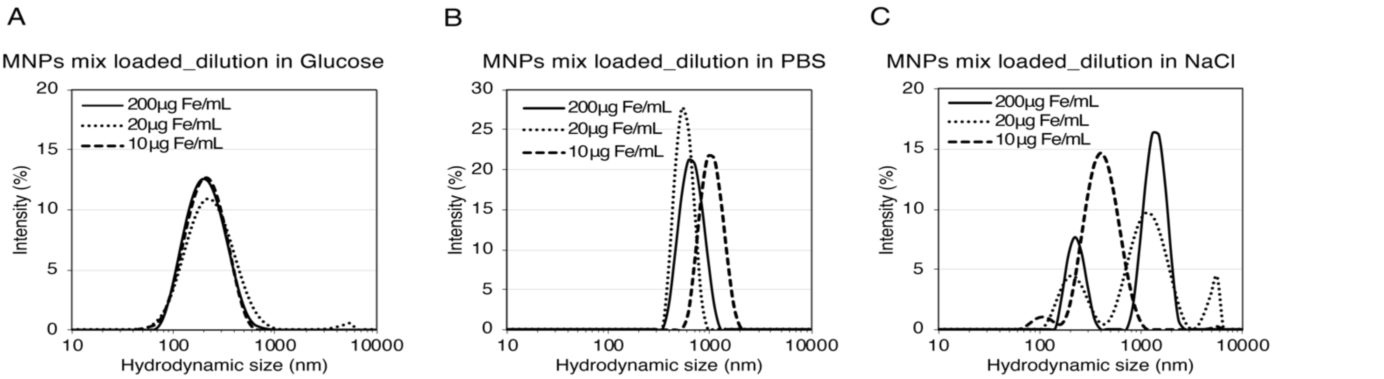

Figure S9. effect of dilution on the hydrodynamic size of MNPs mix by DLS. **(A)** The plots of the hydrodynamic size of MNPs mix loaded with drugs shows dilution up to 100 times does not induce aggregation when Glucose 5% is used. Aggregation occurs rapidly in PBS **(B)** and NaCl **(C)** as shown by the micrometric range of the hydrodynamic size of the MNPs mix.

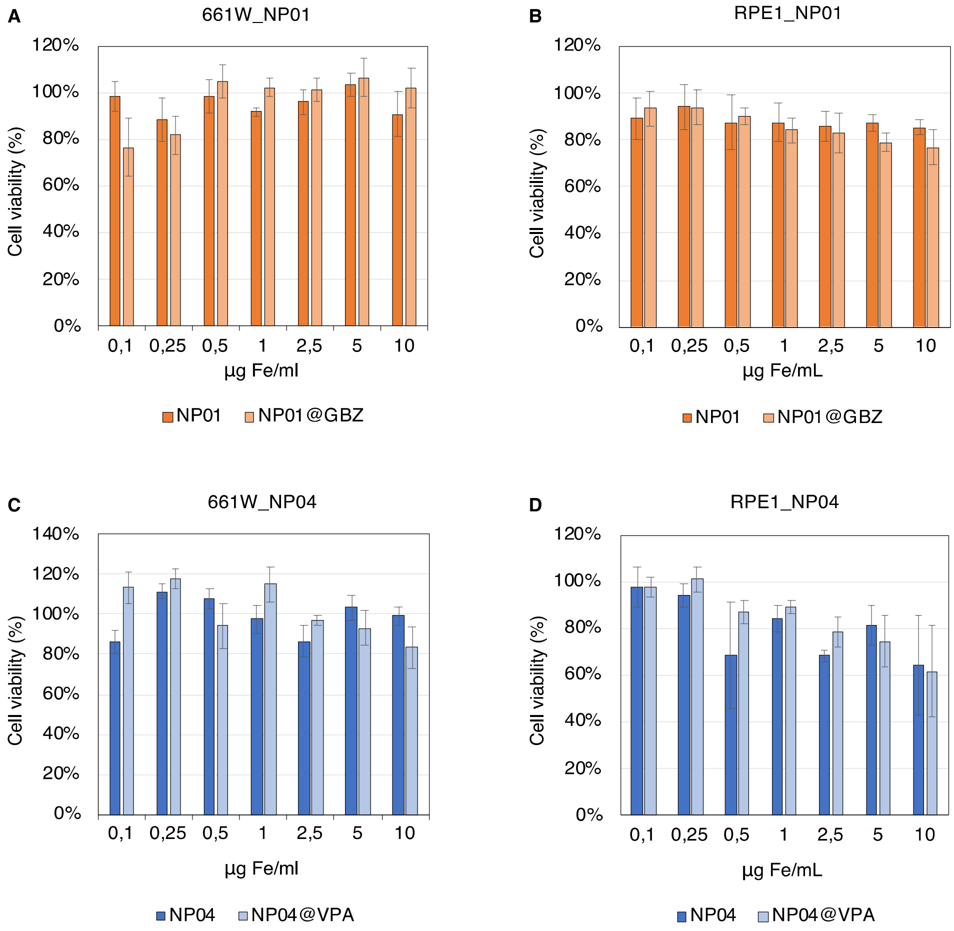

Figure S10. In vitro cell viability of 661W and RPE1 retinal cells treated with increasing concentrations of NP01 and NP04 using OZ Blue assay kit. NP01 when alone (orange bars) or loaded with GBZ (light orange bars) formulated in glucose 5% does not decrease significantly the cell viability of 661W **(A)** and RPE1 **(B)** cells. NP04 when alone (blue bars) or loaded with VPA (light blue bars) formulated in glucose 5% does not decrease significantly the cell viability of 661W cells **(C)** but it has a significant effect on the viability of RPE1 cells **(D)** limited to the highest point in concentration. Mean ± SD ($n = 3$) from three independent experiments are shown. Results are expressed as % using the cells treated with vehicle alone (glucose 5%) as control.

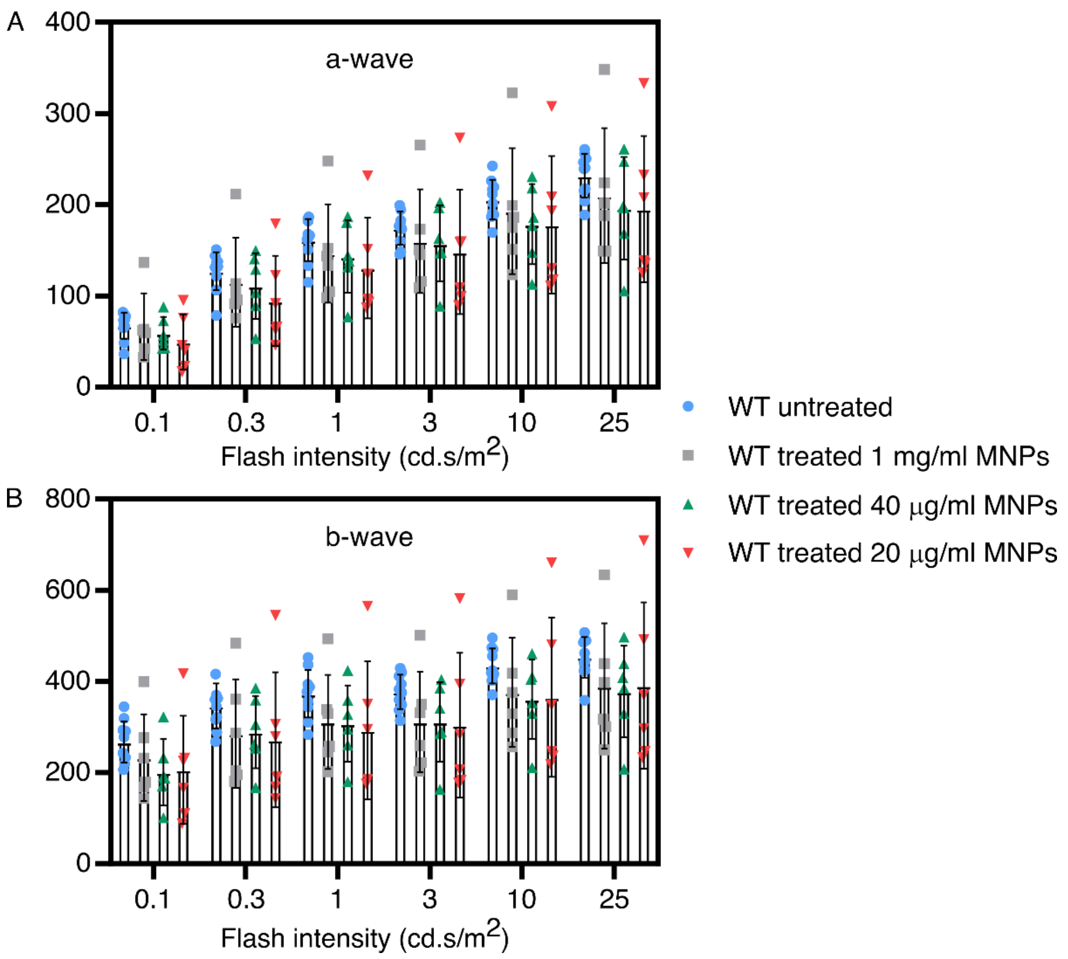

Figure S11. effect of MNPs concentration on ERG recordings in 28 days old *Bbs*$^{+/+}$ mice two weeks after treatment. A-wave **(A)** and b-wave **(B)** amplitudes recorded at increasing flash intensities obtained from WT mice that are not treated (blue circle), treated concentrated MNPs (1 mg Fe/ml, grey square), 1:25 MNPs dilution (40 µg Fe/ml, upward green triangle) and 1:50 dilution (20 µg Fe/ml, downward red triangle). Error bars represent standard error of the mean, for WT untreated group $n = 10$, for all other groups $n = 6$.

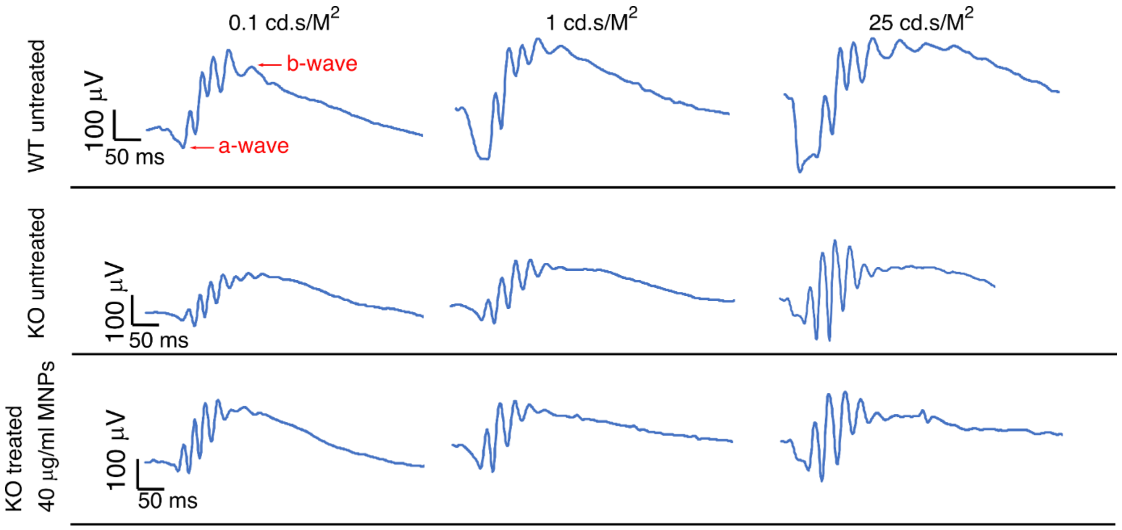

Figure S12. Representative ERG tracings of recordings obtained at increasing flash intensities. The ERG tracing of KO mice is "flattened" compared to WT mice both at minimal (0.1 cd.s/m²) and maximal (25 cd.s/m²) flash intensities. Upon treatment, the a-wave and the b-wave amplitudes improve in KO mice.
